# Larvicidal Potential of *Trattinnickia Burserifolia* Mart. Essential Oil in Controlling the Malaria Vector in the Amazon

**DOI:** 10.3390/ph18050604

**Published:** 2025-04-22

**Authors:** Gisele Guimarães de Oliveira, Stherfany Mac Donald da Silva, Alessandro Pereira de Souza, Leticia Vieira Anchieta da Silva, Anauara Lima e Silva, Ana Cristina Gonçalves Reis de Melo, Rosemary Aparecida Roque, André Correa de Oliveira, Antonio Alves de Melo Filho, Andreimar Martins Soares

**Affiliations:** 1 Bionorte Network Postgraduate Program in Biodiversity and Biotechnology, Federal University of Rondônia, Fundação Oswaldo Cruz, Porto Velho 76801-974, RO, Brazil; andreimar.soares@fiocruz.br; 2 Environmental Chemistry Laboratory, Research and Postgraduate Center in Science and Technology, Federal University of Roraima, Boa Vista 69303-220, RO, Brazil; stherfanymacdonald@gmail.com (S.M.D.d.S.); alessandropsou@gmail.com (A.P.d.S.); leticia.silva@ifrr.edu.br (L.V.A.d.S.); cristina.melo@ufrr.br (A.C.G.R.d.M.); antonio.alves@ufrr.br (A.A.d.M.F.); 3 Multi-User Characterization and Analysis Laboratory, Institute of Research in Drugs and Medicines, Federal University of Paraíba, João Pessoa 58051-900, PB, Brazil; anauaralima@ltf.ufpb.br; 4 Malaria and Dengue Laboratory, Coordination of Society, Environment and Health, National Amazon Research Institute, Manaus 69067-375, AM, Brazil; rosebio1996@yahoo.com.br (R.A.R.); andrebiologo2011@gmail.com (A.C.d.O.)

**Keywords:** *Anopheles*, *Plasmodium*, natural products

## Abstract

**Background:** Among major public health problems, malaria stands out as a tropical disease caused by the *Plasmodium* protozoan, with mosquitoes of the *Anopheles* genus serving as its vectors. This disease affects a significant portion of the population, with the highest incidence in the Legal Amazon, a region responsible for 99% of cases. Although vector control strategies, such as the use of chemical insecticides, are commonly employed, mosquito resistance, environmental impacts, and risks to human health are driving the search for natural alternatives, including the application of essential oils. **Objectives**: This study investigates the larvicidal activity of *Trattinnickia burserifolia* Mart. essential oil against *Anopheles darlingi*. **Methods**: The essential oil was obtained through hydrodistillation, and its chemical composition was identified using gas chromatography–mass spectrometry. The larvicidal assay followed WHO protocols, testing oil concentrations ranging from 20 to 100 µg mL^−1^. **Results**: Efficacy was evaluated after 24, 48, and 72 h to determine LC_50_, LC_90_, and other parameters. Chemical composition analysis revealed the presence of 40 compounds, primarily terpenes such as tricyclene, β-pinene, limonene, and α-pinene, which possess bioactive properties that contribute to vector control. The larvicidal activity test showed that LC_50_ decreased with longer exposure times, indica ting increased efficacy over time. After 72 h, the LC_50_ was 14.51 µg mL^−1^, classifying the essential oil as highly effective. **Conclusions**: Therefore, *T. burserifolia* Mart. essential oil represents a promising natural alternative for malaria vector control.

## 1. Introduction

According to the World Health Organization, it is estimated that 263 million people contracted malaria in 2023 [1]. Data from the Brazilian Ministry of Health highlight that, in the same year, Brazil registered a total of 619,000 deaths caused by the disease [2].

The etiological agent of malaria is a protozoan of the *Plasmodium* genus, with mosquitoes of the *Anopheles* genus serving as its vectors [3]. Malaria is recognized as a serious public health issue and, despite prevention strategies, remains one of the diseases with the greatest impact on morbidity and mortality in tropical regions [4]. Approximately 99% of malaria transmission occurs in the Legal Amazon, which includes nine states: Acre, Amapá, Amazonas, Maranhão, Mato Grosso, Pará, Rondônia, Roraima, and Tocantins [4].

*Anopheles* (Nyssorhynchus) *darlingi* is a key malaria vector. The females can reproduce rapidly in Latin American countries due to favorable climatic conditions [2]. Additionally, anthropized environments such as reservoirs and ponds provide suitable vegetation for the proliferation of this mosquito species [5].

In humans, malaria can be caused by five *Plasmodium* species, with *P. falciparum* and *P. vivax* being the most threatening [4]. The main symptoms include high fever, chills, shivering, sweating, and headache, although clinical manifestations do not always occur [6]. In Brazil, the Ministry of Health has established malaria-related goals: reducing mortality and severe cases, containing incidence, maintaining disease-free areas where transmission has been interrupted, and eradicating malaria from the country [4].

Malaria is considered a medical emergency when it presents severe symptoms (complicated malaria) requiring hospital treatment, which imposes a financial burden on the healthcare system [6].

The most common method for preventing mosquito-borne diseases is vector control, particularly through chemical insecticides such as carbamates, pyrethroids, and organophosphates [7]. However, studies indicate that the continuous use of these chemical insecticides can lead to vector resistance, environmental contamination (water and soil), and toxicity to humans [8,9].

Given these issues, the search for natural products to combat disease vectors has gained prominence [10]. Among these, essential oils have been highlighted for their potential use against disease vectors [11,12,13,14,15,16].

Essential oils are composed of a complex mixture of volatile organic compounds, primarily terpenoids. These lipophilic, often aromatic substances perform distinct biological functions and are derived from the plant’s secondary metabolism [17].

The Amazon hosts the greatest biodiversity in the world, with numerous plant species used for controlling insect-borne diseases [15,16]. The Burseraceae family comprises 18 genera and approximately 700 species, many of which exude resin rich in essential oils [18]. The species investigated in this study is an angiosperm, as evidenced by the presence of fruits and reproductive structures characteristic of this plant group [19]. The resins of the Burseraceae family present, in their composition, tetracyclic and pentacyclic triterpenes, which are secondary metabolites that confer distinctive chemical properties and susceptibility to oxidation [20]. A recent study shows that the genus Trattinnickia has significant pharmacological potential, particularly in relation to anti-inflammatory and analgesic activities, which require further investigation [21]. Within this family, the *Trattinnickia* genus is known for its use in traditional communities for medicinal purposes, such as among the quilombola communities in Oriximiná, Pará, where *Trattinnickia* smoke is used to treat headaches [22].

**Figure 1:**
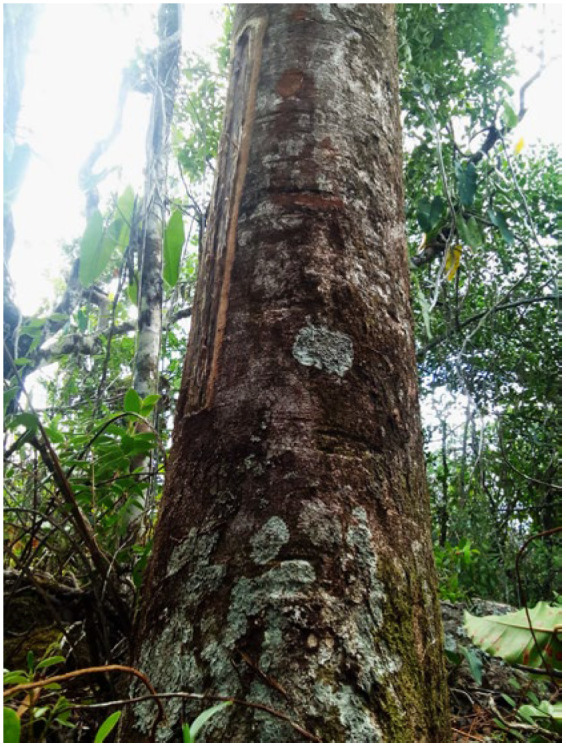
Tree belonging to the Burseraceae family, with morphological characteristics including a DBH (Diameter at Breast Height) of 50 cm, inhabiting clayey soils with an abundant presence of rocks. This angiosperm organism was subjected to an injury process aimed at extracting the essential oil of *T. burserifolia* Mart.

This study aimed to evaluate the larvicidal activity of *Trattinnickia burserifolia* Mart. essential oil (Figure 1) against the malaria vector.

This innovative contribution to the field of public health demonstrates that natural resources from the forest can be utilized for the development of new products and, consequently, for improving the population’s quality of life. It is noteworthy that, in the literature from the past five years, there are scarce records regarding the biological activity of *T. burserifolia* Mart. essential oil in relation to its larvicidal action against *An. darlingi*.

## 2. Results

**Figure 2:**
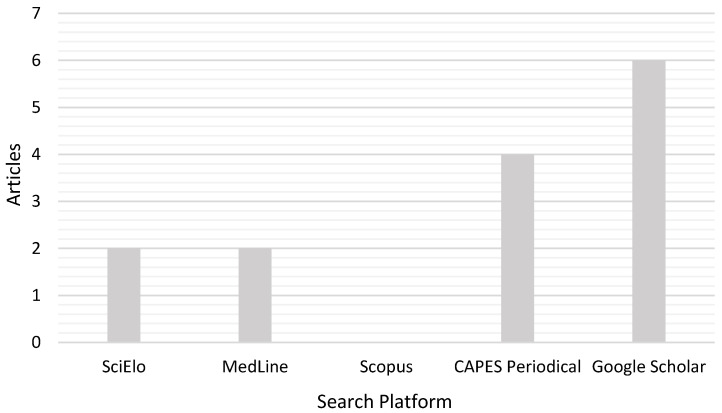
Survey of the main scientific publications on the species under study, conducted on scientific article research platforms, with the exclusion criterion limited to the period from 2020 to 2024.

In the data survey, publications related to the descriptors of this research were identified, as shown in Figure 2.

A few articles were identified as publications related to *T. burserifolia* Mart. essential oil and malaria control, as shown in Table 1.

The publications found provide relevant and updated information on the aspects investigated and contribute to the understanding and analysis of the species.

### 2.1. Characterization by GC-MS

**Figure 3:**
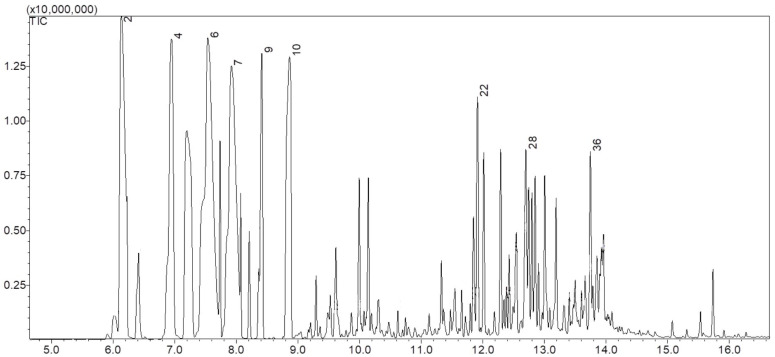
Spectrum obtained from the chemical composition analysis by GC/MS of the essential oil of *T. burserifolia* Mart., highlighting the peaks of the major constituents, which were identified and numbered to facilitate the interpretation of the results.

The essential oil of *T. burserifolia* Mart. exhibited a spectrum with the following peaks, as shown in Figure 3.

A total of 40 constituents were identified in the analysis of the chemical composition of the essential oil of *T. burserifolia* Mart., each contributing specific properties that enhance its application as a larvicide. Among these compounds, the major constituents stand out, representing the largest proportion of the oil’s composition and being responsible for its main pharmacological activities. These constituents are detailed and quantified in Table 2.

### 2.2. Larvicidal Bioassay

The study on the larvicidal activity of *T. burserifolia* Mart. essential oil against the malaria vector *An. darlingi* yielded the following LC_50_ and LC_90_ results, as shown in Table 3, where the data demonstrate the effectiveness of the essential oil in vector control, emphasizing the concentrations required to achieve 50% and 90% larval mortality.

During the experiment, the larvae exhibited slow movements, which, according to the literature, is triggered by the collapse of the central nervous system [24]. This behavior highlights the importance of the relationship between the chemical structure and the biological activity of the compounds, reinforcing the correlation between penetration through the insect’s cuticle and the lipophilicity of the essential oil [25].

## 3. Discussion

The species under study, *T. burserifolia* Mart., showed a scarcity of scientific data from the past five years on major research platforms. A total of 14 publications [21,22,23,24,26,27,28,29,30,31,32,33,34] related to the descriptors established in this research were identified. However, the Scopus platform did not present articles related to the defined descriptors, as shown in Figure 1.

The literature review carried out presented the largest number of publications found [23,24]. Despite this, it is worth noting that only two articles were identified as related to the essential oil of *T. burserifolia* Mart. and malaria control. The most recent article found links between the use of the plant and ecological issues, highlighting its use as an alternative to deforestation. In addition, the author identified another species of the Trattinnickia family [24], listed in Table 1.

The essential oil of *T. burserifolia* Mart. has a very characteristic scent of the plant. During the analysis at the Environmental Chemistry Laboratory of UFRR, the researcher noticed that the oil has a strong and striking aroma, with woody notes, which further highlights its unique characteristics.

The yield of the extracted bioproduct was 0.07%, a value that, although significant, is lower when compared to studies of species belonging to the same family, Burseraceae. Therefore, a higher yield was obtained with other species belonging to this family [28]. Essential oils generally have yields below 1%, adding significant economic value [35]. The data obtained indicate that essential oil yields vary among different plants of the same family, influenced by several factors such as seasonal conditions (season, collection time) and extraction methods used [36,37].

The yield of *T. burserifolia* Mart. essential oil in this study was lower than the results obtained in other research on the same species [38] during the dry season, suggesting relatively stable essential oil production for this species under different climatic conditions. These studies contribute not only to the sustainable development of the plant but also to its potential use in various industries, such as cosmetics, pharmaceuticals, and biopesticide production.

In Brazil, numerous wildfires occurred in 2023, and the state of Roraima stood out as one of the three regions with the highest fire rates in the country. According to data from the National Institute for Space Research (NISR), the state recorded an alarming number of fire outbreaks, and the municipality of Amajari—where the plant material for this study was collected—was among the three most affected municipalities [39]. Research indicates that human activities, such as wildfires, significantly impact the chemical composition of essential oils by rapidly degrading many terpenes, which explains the low yield of *T. burserifolia* Mart. essential oil obtained in this study [40].

The compounds present in the essential oil of *T. burserifolia* Mart. are mainly terpenes, which are distinguished by their pharmacological effects. These compounds are widely used by indigenous populations for various purposes, including healthcare and traditional practices. Terpenes play a fundamental role in folk medicine due to their antimicrobial, anti-inflammatory, and antioxidant properties [38]. The use of these essential oils contributes to the development of new treatments for tropical diseases such as malaria [41].

Gas chromatography–mass spectrometry (GC-MS) analysis revealed a complex chemical profile with the identification of 40 compounds (Table 2). Among the major constituents, tricyclene (15.86%) stands out as a molecule of growing scientific interest due to its therapeutic potential. Recent studies associate tricyclene with antifungal and anti-inflammatory activities [42], highlighting its role as a promising bioactive compound. Other monoterpenes, such as β-pinene (14.21%), limonene (12.23%), α-pinene (9.38%), and α-terpinene (6.64%), also contributed significantly to the composition of the essential oil, in agreement with previous studies [38].

In the essential oil of *T. burserifolia* Mart., ten major chemical constituents were identified, and these are highlighted in Table 4. The remaining compounds were excluded from the analysis due to their low area values in the chromatogram, which did not reach significance for the study. This approach prioritized the most relevant components, ensuring a precise analysis focused on the main active constituents.

Despite their great importance in the scientific field, bioprospecting studies remain underexplored in Brazil [66]. Scientific databases are crucial for obtaining technological and scientific information, as the development of new drugs depends on the data provided by these studies [67].

In the larvicidal activity assay, the concentration required to cause 50% larval mortality (LC_50_) decreased with increased exposure time, as shown in Table 3. After 24 h, the LC_50_ was 96.35 µg mL^−1^, classified as toxic. However, after 72 h, the LC_50_ decreased to 14.51 µg mL^−1^, indicating even greater activity. These results suggest that prolonged exposure to *T. burserifolia* Mart. essential oil enhances its effects on larvae [68].

Essential oils contain a variety of secondary metabolites with insecticidal potential, serving as an alternative to synthetic products. These complex mixtures of bioactive compounds reduce the risk of insect resistance [41].

Mosquito larvae control can be achieved through natural compounds such as essential oils. Monoterpenes, such as α-pinene, present in some plants, have demonstrated strong larvicidal activity [69]. Additionally, oleic acid acts on the insect’s body wall, inhibiting essential enzymes for its development. These mechanisms of action suggest the potential of these compounds in controlling disease vectors such as malaria [70].

The larvicidal action of *T. burserifolia* Mart. essential oil occurred within 24 h, with the observation of the larvae’s slow behavior, whereas another study observed larvicidal activity after 48 h [71]. The results of this research are in line with another study, which analyzed essential oils from the Burseraceae family and identified β-pinene, α-terpineol, and caryophyllene as key components effective against *Anopheles* larvae [72].

Some essential oils have a sublethal effect on mosquito larvae; that is, they have an inhibitory effect on the development of the mosquito, without necessarily causing death. This characteristic is important for malaria control, as it allows action in the early stages of the mosquito’s life cycle before it becomes capable of transmitting the disease [73].

The presence of monoterpenes and sesquiterpenes, identified in research on Piperaceae essential oil [74], was also detected in *T. burserifolia* Mart. essential oil, as shown in Figure 2 and Table 2 [38].

The literature reveals that the action of terpenes, identified as chemical constituents of essential oils, enhances insecticidal activity [25,75,76]. An ethnobotanical study aimed at listing plant species used to treat malaria identified *T. burserifolia* Mart. as one of the most effective species in treating the disease [23], highlighting the potential of *T. burserifolia* Mart. essential oil extracted from the bark of its trunk in malaria control and beyond.

## 4. Materials and Methods

A preliminary systematic review was conducted using the following platforms: CAPES Journals, Google Scholar, Scopus, MedLine, and SciELO. The selected descriptors were Malaria, essential oil, and *T. burserifolia* Mart. Articles published between 2020 and 2024 were included in this study, while those published before this period were excluded.

### 4.1. Recording and Collection of the Species

The authorization for the collection of the plant species *T. burserifolia* Mart. was obtained through the Biodiversity Authorization and Information System (SISBIO) under permit No. 93967-1 and registered in the National System for Genetic Heritage and Associated Traditional Knowledge Management (SisGen) under registration No. A918C0D, in compliance with Law No. 13.123/2015 and its regulations.

The plant material (stem bark) of *T. burserifolia* Mart. was collected in the Tepequem Mountain Range, in the municipality of Amajari, Roraima (3°46′11.4″ S 61°43′18.8″ W), between September and March 2024, a period following wildfires in the region. Taxonomic identification was confirmed by comparison with a previously identified specimen deposited in the herbarium of the Federal University of Roraima (UFRR) under voucher numbers 3863 and 4873 to 4921.

### 4.2. Essential Oil Extraction

The stem bark (1 kg) of *T. burserifolia* Mart. was crushed and placed in a round-bottom flask with 4 L of water for the hydrodistillation process. The essential oil was isolated using a Clevenger apparatus adapted with a double condenser for 3 h continuous [77].

### 4.3. Identification of Chemical Constituents by Gas Chromatography Coupled withMass Spectrometry

The essential oil of *T. burserifolia* Mart. was diluted in HPLC-grade hexane (≥95%) Biograde (BioScie Industry, Anápolis, Brazil). The diluted solution was injected into a gas chromatograph coupled with mass spectrometry (GC-MS). The analysis was performed using a Shimadzu^®^ GCMS-QP2010 Ultra (Kyoto, Japan), equipped with an RTX-5MS capillary column (5% diphenyl/95% dimethyl polysiloxane) (30 m × 0.25 mm × 0.25 µm). The injection volume was 1 µL, with a split ratio of 1:50.

The chromatographic conditions were as follows: sample inlet temperature of 180 °C, injector temperature of 280 °C, and oven temperature initially set at 40 °C, maintained for 2 min, then increased gradually (10 °C/min) to 100 °C and subsequently to 280 °C. Helium was used as the carrier gas. Electron impact ionization was applied at 70 eV, with full scan mode covering a mass range of 35–400 *m*/*z* [78]. Chromatographic data were processed using Shimadzu GC-MS software (GC-MS Solution, Version 4.20) and compared with reference spectra available in the Shimadzu software database and the National Institute of Standards and Technology (NIST) library (NIST), as well as in other research [46].

### 4.4. Collection of An. Darlingi Larvae

The larvae were provided by the Malaria and Dengue Laboratory of the National Institute for Amazonian Research (INPA/Manaus) in February 2024. They were collected from the Puraquequara community at the D’Chagas site (3°08′42″ S 60°17′26″ W) in Manaus, Amazonas, Brazil. The larvae were found in a fish farming tank with permanent, clear water. The environment featured horizontal vegetation, rich in organic matter, with the presence of algae.

### 4.5. Larvicidal Assay

**Figure 4:**
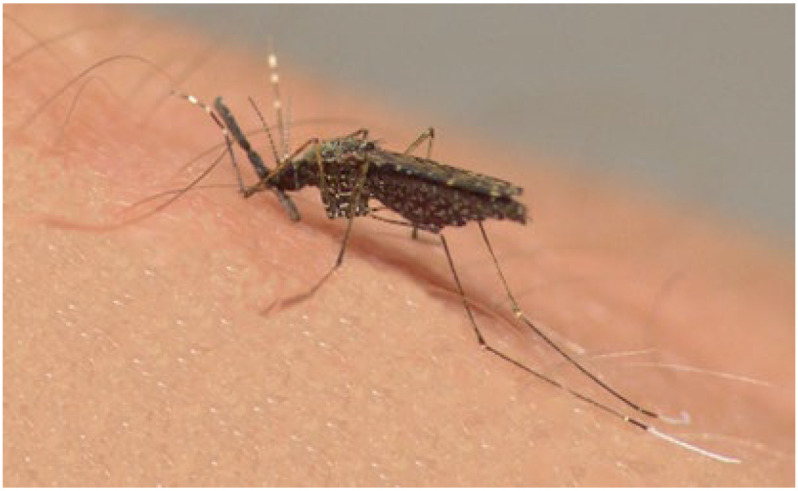
Representation of the mosquito *An. darlingi*, the vector of *Plasmodium* spp., the parasite that causes malaria. The image emphasizes the importance of controlling the Anopheles vector at the larval stage as a fundamental strategy to interrupt malaria transmission.

The bioassay was conducted using third-instar larvae of the *An. darlingi* mosquito (Figure 4), following the World Health Organization (WHO) protocol [79]. The tested concentrations, each with three replicates, were prepared in dimethyl sulfoxide (DMSO), which was used as a negative control, while α-cypermethrin served as the positive control. The larvae of *An. darlingi* were distributed in plastic cups (180 mL) containing 100 mL of water and concentrations ranging from 20 to 100 µg mL^−1^. This concentration range was selected based on the guidelines of the World Health Organization (WHO) for larvicidal tests. These guidelines recommend the use of scaled concentrations to determine toxicity parameters, such as lethal concentrations (LC_25_, LC_50_, and LC_100_) [74].

The percentage of activity at each concentration was calculated after 24, 48, and 72 h, according to Equation (1).

(1) $$\mathrm{Larvicidal}\;\mathrm{Activity}\%=\frac{number\;of\;dead\;larvae}{total\;number\;of\;larvae}\times 100\%$$

### 4.6. Statistical Analysis

The Probit analysis was performed using IBM^®^ SPSS^®^ Statistics software (Version 9.0) to estimate LC_50_ and LC_90_ values, linear equations, chi-square (χ^2^), and degrees of freedom [80].

## 5. Conclusions

The essential oil of *T. burserifolia* Mart. has demonstrated significant potential as an effective larvicide for controlling the malaria vector, *An. darlingi*. However, the limited number of records found on research platforms regarding the larvicidal activity of this essential oil highlights the need for further exploration of this promising natural product. This study has emphasized the ecological and pharmacological value of the species, particularly in the Amazon biome, where the plant and the malaria vector coexist.

The variability in the chemical composition of *T. burserifolia* Mart. essential oil, influenced by seasonal and environmental factors, may impact its larvicidal efficacy. Therefore, future research should focus on optimizing the formulation of the essential oil for practical applications, exploring synergistic effects with other bioactive compounds, and evaluating its efficacy against other mosquito vectors.

This study contributes to the development of sustainable, plant-based larvicides that can be integrated into vector management strategies, strengthening malaria control initiatives. Additionally, it paves the way for further investigations into the pharmacological and ecological potential of *T. burserifolia* Mart., reinforcing the importance of conserving Amazonian biodiversity.

## Figures and Tables

**Table 1:** Publications obtained in the bibliographic survey related to the species under study, which are within the period previously selected for this research.

Article Title	Author, Journal and Year
Ethnobotanical treatment of tropical diseases prescribed by practitioners and the bioenergetic profile of the population involved in southern Amazonas.	[23]
Diversity of *Plasmodium* vectors and functional traits of trees in a disturbed forest in Tingo María, 2022.	[24]

**Table 2:** Major constituents identified in the essential oil of *T. burserifolia* Mart., represented by the peaks shown in Figure 2, properly numbered and associated with their respective properties and chemical profile characterization.

Corresponding Peak	Constituents	Molecular Formula	Retention Time (min)	Area (%)
2	α-Pinene	C_10_H_16_	6.133	9.38
4	β-Pinene	C_10_H_16_	6.948	14.21
6	Tricyclene	C_10_H_16_	7.537	15.86
7	Limonene	C_10_H_16_	7.925	12.23
9	γ-Terpinene	C_10_H_16_	8.415	3.83
10	α-Terpinene	C_10_H_16_	8.867	6.84
22	α-Cubebene	C_15_H_24_	11.917	3.58
28	α-Bergamotene	C_15_H_24_	12.702	3.50
29	α-Muurolene	C_15_H_24_	12.852	2.82
36	δ-Cadinol	C_15_H_26_O	13.749	2.00
-	Others Total	-	-	25.75 100.00

**Table 3:** Results obtained from toxicity assays with *An. darlingi* larvae, highlighting the LC_50_, LC_90_ values, confidence interval (CI), and linear regression equations associated with the larvicidal activity of the essential oil of *T. buserifolia* Mart.

Time (h)	LC_50_ (µg mL^−1^) (LCL-LCU)	LC_90_ (µg mL^−1^) (LCL-LCU)	χ^2^ (Df)	Linear Equation	RP
24	96.35 (68.714–289.888)	366.78 (170.175–471.410)	0.079 (3) *	Y =−4.380 + 1.353x	0.003
48	27.74 (2.691–43.715)	189.35 (94.024–371.725)	5.011 (3) *	Y =−2.236 + 1.066x	0.011
72	14.51 (2.784–35.984)	77.87 (49.174–831.514)	3.810 (3) *	Y =−2.041 + 1.176x	0.022
α-Cypermethrin	0.33 (0.313–0.357)	0.60 (0.565–0.668)	0.03 (4) *	4.950 ± 0.351	1

LC_50_ and LC_90_—Concentration limits required to kill 50% and 90% of the larvae. LCL—lower confidence limit at 95%. LCU—upper confidence limit at 95%. **χ**^2^—non-significant chi-square (*p* < 0.05). Df—degrees of freedom. RP—relative potency = LC_50_ α-cypermethrin/LC_50_ of the natural product. * *p* ≤ 0.05 (significant).

**Table 4:** Description of the major constituents identified in the essential oil of *T. burserifolia* Mart., including their chemical class and main actions and benefits.

Chemical Constituent	Chemical Grade	Share
Tricyclene	Monoterpene with three additional methyl substituents [43]	Potential pharmacological activity against protozoa of the genus *Plasmodium* [44].
β-Pinene	Monoterpene, isomer of pinene with an exocyclic double bond [45,46]	It has larvicidal activity. It has a role as a plant metabolite [47].
Limonene	Substituted monoterpene [46,48]	One of the most commonly found compounds in plant species. It is a monoterpene that has larvicidal potential. It plays a role as a human metabolite [49].
α-Pinene	Methyl-substituted monoterpenes [50]	Anxiolytic, antioxidant, anti-inflammatory, analgesic, antimicrobial, and anticoagulant effects. It has a role as a plant metabolite [51].
α-Terpinene	Isomeric monoterpene [52]	It has biological activities, including high therapeutic potential. It has a role as a volatile oil component and a plant metabolite [53,54].
γ-Terpinene	Isomeric monoterpenes [55]	Catalysis process. Antimicrobial activity. Has a role as an antioxidant and a plant metabolite. A volatile component of the oil is a human xenobiotic metabolite [56,57].
α-Cubebene	Tricyclic sesquiterpene [58]	It has fungicidal action. It has a role as a plant metabolite. A human metabolite is a volatile oil component [59].
α-Bergamotene	Sesquiterpene [60]	Used in biosynthesis of products. It has a role as a plant metabolite and a volatile oil component [61].
α-Muurolene	Sesquiterpene [62]	Insecticidal action [63].
δ-Cadinol	Cadinane sesquiterpenoid [64]	Antimicrobial activity. It has a role as an algal metabolite and a plant metabolite [65].

## Data Availability

Data is contained within the article or Supplementary Materials.

## Supplementary Materials

The following supporting information can be downloaded at https://www.mdpi.com/article/10.3390/ph18050604/s1: Figure S1: Espectro do Triciclene (Adams, 2007) [46]; Figure S2: Espectro do β-Pineno (Adams, 2007) [46]; Figure S3: Espectro do Limoneno (Adams, 2007) [46]; Figure S4: Espectro do α-Pineno (Adams, 2007) [46]; Figure S5: Espectro do α-Terpineno (Adams, 2007) [46]; Table S1. Publications found in scientific databases containing the terms ‘Trattinnickia’ and ‘Malaria’ between 2020 and 2024.

## References

[1] WHO World Health Organization. Malária.

[2] Brazil Ministry of Health Epidemiological Bulletin—Epidemiological Overview of Malaria in 2021: Seeking the Path to Malaria Elimination in Brazil. https://www.gov.br/saude/pt-br/assuntos/saude-de-a-a-z/m/malaria.

[3] Veronesi R., Focaccia R. (2021). Treatise on Infectious Diseases.

[4] Ministry of Health (2021). Health Surveillance Secretariat. Department of Immunization and Communicable Diseases. Malaria Treatment Guide in Brazil.

[5] dos Santos F., Xu M., Bravo de Guenni L., Lourenço-de-Oliveira R., Rubio-Palis Y. (2024). Characterization of larval habitats of *Anopheles (Nyssorhynchus) darlingi* and associated species in malaria areas in western Brazilian Amazon. Mem. Inst. Oswaldo Cruz.

[6] Barreto T.M.A.C., Ferko G.P.S., Rodrigues F.S. (2022). Hospital Costs of Diseases Attributable to Environmental Factors among Residents of Boa Vista and the Increase in Care for Venezuelan Migrants. Cad. Saúde Coletiva.

[7] Adhikari K., Khanikor B., Sarma R. (2022). Persistent Susceptibility of *Aedes aegypti* to Eugenol. Sci. Rep..

[8] Medeiros J.F.D., Acayaba R.D.A., Montagner C.C. (2021). Chemistry in the Assessment of Human Health Impact from Pesticide Exposure. Química Nova.

[9] Mossa A.T., Mohafrash S., Chandrasekaran N. (2018). Safety of Natural Insecticides: Toxic Effects on Experimental Animals. BioMed Res. Int..

[10] Vale E.S.M., Rodrigues I.B., Tadei W.P., Andrade J.K.B. (2024). Evaluation of the Use of Plant Extracts in Aedes spp. Breeding Sites on the Campus of the National Institute for Amazonian Research (INPA), Manaus, Amazonas. Fundamentals and Research in Environmental and Agricultural Sciences.

[11] Souza A.L.D.C., Rebouças C.K.D.O., Albuquerque C.C.D., Moura C.D.C.F.L., Torres T.M., Batista J.I.L., Bezerra A.C.D.S. (2020). In Vitro Anthelmintic Activity of *Lippia gracilis* Schauer Essential Oil Against Egg-Hatching of Goat Gastrointestinal Nematodes. Arq. Inst. Biológico.

[12] Torres-Santos P.T., Farias I.F., Almeida M.D., Passos G.S., Ribeiro L.A., Rolim L.A., Horta M. (2021). Acaricidal Efficacy and Chemical Study of Hexane Extracts of the Leaves of *Neoglaziovia variegata* (Bromeliaceae) Against the Tick Rhipicephalus microplus. Exp. Appl. Acarol..

[13] Sampaio M.G.V., Bezerra Dos Santos C.R., Cortez Sombra Vandesmet L., Souza Dos Santos B., Bianca Da Silva Santos I., Correia M.T.D.S., Da Silva M.V. (2021). Chemical Composition, Antioxidant, and Antiprotozoal Activity of *Eugenia gracillima* Kiaersk. Leaves Essential Oil. Nat. Prod. Res..

[14] Alcantara I.S., Martins A.O.B.P.B., de Oliveira M.R.C., Coronel C., Gomez M.C.V., Rolon M., de Menezes I.R.A. (2021). Cytotoxic Potential and Antiparasitic Activity of the Croton rhamnifolioides Pax Leaves. & K. Hoffm. Essential Oil and Its Inclusion Complex (EOCr/β-CD). Polym. Bull..

[15] Pereira J.R., da Silva S.M.P., Marques M.O.M. (2022). Efficacy of Essential Oil of *Lippia sidoides* (Verbenaceae) for Controlling the Cattle Tick *Rhipicephalus* (Boophilus) *microplus* on Naturally Parasitized Animals under Field Conditions. Vet. Parasitol..

[16] Gomes G.A., Rondon F.C.M.R.M., Carneiro-Torres D.S.C.T., Fampa P.F., Bevilaqua C.M.L.B.L., Bandeira P.N.B., de Carvalho Carvalho M.G. (2022). *Croton pulegiodorus* Baill and *Croton piauhiensis* Mull. Arg. (Euphorbiaceae) Essential Oils: Chemical Composition and Anti-Leishmania Activity. Rev. Virtual Química.

[17] Bieski I.G.C., Santos J.L.U.D., Ferreira M.D.L., Garcia P.C., Dourado S.H.A., Januário A.B., Apolinário J.M.D.S.D.S. (2020). Economic and Therapeutic Potential of the Most Used Essential Oils in Brazil. Rev. Fitos.

[18] Daly D.C., Perdiz R.O., Fine P.V.A., Damasco G., Martínez-Habibe M.C., Calvillo-Canadell L. (2022). A Review of Neotropical Burseraceae. Braz. J. Bot..

[19] Thulin M., Beier B.A., Razafimandimbison S.G., Banks H.I. (2008). *Ambilobea*, a new genus from Madagascar, the position of *Aucoumea*, and comments on the tribal classification of the frankincense and myrrh family (Burseraceae). Nord. J. Bot..

[20] Passero L.F., Laurenti M.D., Santos-Gomes G., Soares Campos B.L., Sartorelli P., Lago J.H. (2014). Plants used in traditional medicine: Extracts and secondary metabolites that exhibit antileishmanial activity. Curr. Clin. Farmacol..

[21] de Souza A.A., Ortíz B.L.S., de Carvalho Rocha Koga R., Sales P.F., da Cunha D.B., Guerra A.L.M., Carvalho J.C.T. (2021). Secondary Metabolites Found among the Species *Trattinnickia rhoifolia* Willd. Molecules.

[22] Albino R.C., Braz M.M., Bizzo H.R., Santana da Silva R.V., Leitão S.G., Ribeiro de Oliveira D. (2021). Amazonian Medicinal Smokes: Chemical Analysis of Burseraceae Pitch (Breu) Oleoresin Smokes and Insights into Their Use on Headache. J. Ethnopharmacol..

[23] Larocca D.G., Júnior N.G.R., Vicente R.E., da Silva I.V. (2021). Ethnobotanical Treatment of Tropical Diseases, Malaria and Dengue, Prescribed by Bioenergético Practitioners and Profile of the Involved Population in Meridional Amazon. Etnobiología.

[24] Cierto L.E.O., Zevallos A.W., Valencia-Reyes Z.L., Vilchez-Ochoa G.L., Salas-Zeballos V.R., Curo G.G., Dumont J.R.D. (2022). Diversity of Plasmodium Vectors and Functional Traits of Trees in the Disturbed Forest in Tingo María. Bol. Malariol. Environ. Health.

[25] Isman M.B. (2006). Botanical insecticides, deterrents, and repellents in modern agriculture and an increasingly regulated world. Annu. Rev. Entomol..

[26] Batista L., Stangerlin D.M., Melo R.R.D., Souza A.P.D., Silva E.D.S., Pariz E. (2021). Mechanical resistance and chemical characterization of Amazonian deteriorated woods in field tests. Madera Bosques.

[27] Silva E.D.S., Júnior E.A.B., Silva G.A.D.O., Curvo K.R., Stangerlin D.M., Melo R.R.D., Souza A.P.D. (2022). Surface deterioration of five amazonian wood exposed to natural weathering. Madera Bosques.

[28] de Souza A.A., Ortíz B.L.S., Borges S.F., Pinto A.V.P., Ramos R.D.S., Pena I.C., Tavares Carvalho J.C. (2022). Acute Toxicity and Anti-Inflammatory Activity of *Trattinnickia rhoifolia* Willd (Sucuruba) Using the *Zebrafish* Model. Molecules.

[29] Bimestre T.A., Silva F.S., Tuna C.E., dos Santos J.C., de Carvalho J.A., Canettieri E.V. (2023). Physicochemical Characterization and Thermal Behavior of Different Wood Species from the Amazon Biome. Energias.

[30] Magalhães L.M. (2017). Identification of Flavonoids by LC-MS/MS in Leaf Extract of *Trattinnickia rhoifolia* (Willd) and Evaluation of Antioxidant Activity. Master’s Dissertation.

[31] Canetti A., Braz E.M., de Mattos P.P., Basso R.O., Filho A.F. (2021). A novel approach to maximize wood production in sustainable management of the Amazon rainforest. Ann. For. Sci..

[32] Delascio-Chitty F., Brewer-Carías C. (2023). Aspectos generales de la vegetación, flora y plantas útiles del valle del río Kanarakuni, Alto río Caura, estado Bolívar, Venezuela. Mem. Fund. La Salle Cienc. Nat..

[33] Muñoz-Romo M., Ramoni-Perazzi P. (2020). Unraveling the leaf-dropping behavior behind bat folivory: Do bats use biological control against roost parasites?. Mammalia.

[34] Silveira Z.D.S., Macêdo N.S., Bezerra S.R., Siyadatpanah A., Coutinho H.D.M., Seifi Z., Balbino V.D.Q. (2022). Phytochemistry and Biological Activities of *Amburana cearensis* (Allemão) ACSm. Molecules.

[35] Asbahani E.A. (2015). Essential oils: From extraction to encapsulation. Int. J. Pharm..

[36] Schindler B., Silva D.T.D., Heinzmann B.M. (2018). Effect of seasonality on the essential oil yield of *Piper gaudichaudianu* m kunth. Cienc. Florest..

[37] Ribeiro M.S., Bonilla H.O., Lucena P.M.E. (2018). Influence of Seasonality and the Circadian Cycle on the Yield and Chemical Composition of Essential Oils from Croton spp. of the Caatinga. Iheringia Série Botânica.

[38] de Oliveira G.G., Da Silva H.B., Vital E.S. (2014). Chemical constituents and antimicrobial activity of essential oil of *Trattinnickia burserifolia,* Tepequém, Roraima, Brazil. Planta Med..

[39] Brazil INPE—National Institute for Space Research TerraBrasilis Platform: Comparative Annual Table of Brazilian States in 2023. https://terrabrasilis.dpi.inpe.br/queimadas/situacao-atual/situacao_atual/.

[40] Hassiotis C.N. (2010). Chemical compounds and essential oil release through decomposition process from *Lavandula stoechas* in Mediterranean region. Biochem. Syst. Ecol..

[41] Alsalhi M.S., Elumalai K., Devanesan S., Govindarajan M., Krishnappa K., Maggi F. (2020). The aromatic ginger *Kaempferia galanga* L. (Zingiberaceae) essential oil and its main compounds are effective larvicidal agents against *Aedes vittatus* and *Anopheles maculatus* without toxicity on the non-target aquatic fauna. Ind. Crops Prod..

[42] dos Anjos R.C.L., Cortes B.A.G., Alves E.M., de Farias L.H.M., de Melo Cazal C., Pereira P.S., Christofoli M. (2022). Chemical Composition and Antifungal Activity of the Essential Oil from the Flowers of *Bauhinia forficata* (Link) and Its Properties on the Germination of *Cucurbita maxima* (Duchesne) Seeds. Res. Soc. Dev..

[43] National Library of Medicine (EUA), National Center for Biotechnology Information PubChem Resumo do Composto Para CID 79035, Tricyclene. https://pubchem.ncbi.nlm.nih.gov/compound/Tricyclene.

[44] de Andrade F.F., da Silva A.V.C., de Araújo L.M., da Silva L.G.M., da Silva J.K., da Silva R.N.A. (2025). Essential oils from the Amazon: Potential and future perspectives for Andiroba, Breu-Branco, Buriti, and Copaíba. Stud. Eng. Exact Sci..

[45] National Library of Medicine (EUA), National Center for Biotechnology Information PubChem Resumo do Composto Para CID 14896, beta-Pinene. https://pubchem.ncbi.nlm.nih.gov/compound/beta-Pinene.

[46] Adams R.P. (2007). Identification of Essential Oil Components by Gas Chromatography/Mass Spectrometry.

[47] Santana R.C., dos Santos Rosa A., da Silva Mateus M.H., Soares D.C., Atella G., Guimaraes A.C., Pinto-da-Silva L.H. (2020). In vitro leishmanicidal activity of monoterpenes present in two species of Protium (Burseraceae) on *Leishmania amazonensis*. J. Ethnopharmacol..

[48] National Library of Medicine (EUA), National Center for Biotechnology Information PubChem Resumo do Composto Para CID 22311, Limonene, (+/-). https://pubchem.ncbi.nlm.nih.gov/compound/Limonene.

[49] de Santana B.P., de Deus R.G., de Oliveira O.G.L., Vieira T.M., Roner M.N.B. (2022). Óleos essenciais com atividade carrapaticida contra *Rhipicephalus* (Boophilus) *microplus* e *Rhipicephalus sanguineus*: Uma revisão. Res. Soc. Dev..

[50] National Library of Medicine (EUA), National Center for Biotechnology Information PubChem Resumo do Composto Para CID 6654, alfa-PINENO. https://pubchem.ncbi.nlm.nih.gov/compound/alpha-PINENE.

[51] da Silva R.C.S., de Souza Arruda I.R., Malafaia C.B., de Moraes M.M., Beck T.S., da Camara C.A.G., Machado G. (2022). Synthesis, characterization and antibiofilm/antimicrobial activity of nanoemulsions containing *Tetragastris catuaba* (Burseraceae) essential oil against disease-causing pathogens. J. Drug Deliv. Sci. Technol..

[52] National Library of Medicine (EUA), National Center for Biotechnology Information PubChem Resumo do Composto Para CID 7462, alfa-Terpineno. https://pubchem.ncbi.nlm.nih.gov/compound/alpha-Terpinene.

[53] Umeh N.E., Onuorah R.T., Ekweogu C.N., Ijioma S.N., Egeduzu O.G., Nwaru E.C., Ugbogu E.A. (2024). Chemical profiling, toxicity assessment, anti-diarrhoeal, anti-inflammatory and antinociceptive activities of *Canarium schweinfurthii* Engl. (Burseraceae) bark in rats. J. Ethnopharmacol..

[54] Acikgul F.C., Duran N., Kutlu T., Ay E., Tek E., Bayraktar S. (2024). The therapeutic potential and molecular mechanism of Alpha-pinene, Gamma-terpinene, and P-cymene against melanoma cells. Heliyon.

[55] National Library of Medicine (EUA), National Center for Biotechnology Information PubChem Resumo do Composto Para CID 7461, gama-Terpineno. https://pubchem.ncbi.nlm.nih.gov/compound/gamma-Terpinene.

[56] Hernández-Ruiz R., Solas M., Suárez-Pantiga S., Pedrosa M.R., Sanz R. (2025). γ-Terpinene: Biorenewable Reductant for the Molybdenum-Catalyzed Reduction of Sulfoxides, *N*-Oxides and Nitroarenes. Adv. Synth. Catal..

[57] de Castro Borba E.R., Mubárack T.C., Luz T.R.S.A., Silveira D.P.B., Silva A.Z., Figueiredo P.D.M.S., Coutinho D.F. (2021). Chemical characteristics and antimicrobial activity of the essential oil of *Plectranthus amboinicus* (lour.) Spreng. cultivated with different fertilizers. Res. Soc. Dev..

[58] National Library of Medicine (EUA), National Center for Biotechnology Information PubChem Resumo do Composto Para CID 6431138, 6-epi-alpha-Cubebene. https://pubchem.ncbi.nlm.nih.gov/compound/6-epi-alpha-Cubebene.

[59] Jaramillo-Colorado B.E., Arroyo-Salgado B., Palacio-Herrera F.M. (2025). Antifungal activity of four *Piper genus* essential oils against postharvest fungal Fusarium spp. isolated from *Mangifera indica* L. and *Persea americana* Mill. fruits. Ind. Crops Prod..

[60] National Library of Medicine (EUA), National Center for Biotechnology Information PubChem Resumo do Composto Para CID 86608, alfa-Bergamoteno. https://pubchem.ncbi.nlm.nih.gov/compound/alpha-Bergamotene.

[61] Wen Y.H., Chen T.J., Jiang L.Y., Li L., Guo M., Peng Y., Zhu P. (2022). Unusual (2R,6R)-bicyclo [3.1.1] heptane ring construction in fungal *α-trans*-bergamotene biosynthesis. Iscience.

[62] Lima S.C., Oliveira A.C., Costa M.L.L., Abensur D.D., dos Santos Andrade A.T., Souza H.V., Roque R.A. Larvicidal Effect and Mechanism of Action of the Essential Oil and Major Compound from Piper brachypetiolatum (Piperaceae) Against Aedes aegypti (Linnaeus, 1762) Larvae, with Protection of Non-Target Aquatic Animals. 2024. [S.l.: S.n.]. https://www.researchsquare.com/article/rs-5233897/v1.

[63] Mendonça J.F., Sousa A.H.D., Faroni L.R., Fernandes C.C., Santos A.C.D., Lopes L.M., Prates L.H. (2024). Yield, composition and toxicity of piperaceae volatiles to pest insects. Rev. Caatinga.

[64] National Library of Medicine (EUA), National Center for Biotechnology Information PubChem Resumo do Composto Para CID 3084311, delta-Cadinol. https://pubchem.ncbi.nlm.nih.gov/compound/delta-Cadinol.

[65] Veloso C.A.G., de Souza P.H.S., Nóbrega F.P., de Medeiros A.C.D., Fechine I.M., de Melo J.I.M., Tavares J.F. (2020). Chemical composition of *Varronia dardani* (Taroda) J.S. Mill essential oil and its antibiofilm activity. Braz. J. Dev..

[66] Carvalho R.A., da Conceição Lima A.M., Pereira Á.I.S., Sobrinho O.P.L., Ribeiro F.A.A., da Silva Costa S.T., Lopes T.Y.A. (2020). Pharmacological Potentials of Aloe Vera: A Study Conducted Using Scientific and Technological Prospecting Techniques. Cad. Prospecção.

[67] Pereira S.A., de Mendonça M.S., Barbalho C.R.S., de Alencar M.S.M., de Souza C.D.M. (2015). Scientific and Technological Prospecting of the *Genus jatropha* (Euphorbiaceae). Cad. Prospecção Salvador.

[68] Barroso J.A. (2022). Evaluation of the Insecticidal Activity Potential of Essential Oils Extracted from *Azadirachta indica* and *Ricinus communis* L. on *Anopheles* spp. and *Aedes* spp. Larvae. Rev. Ibero-Am. Humanidades Ciências Educ..

[69] Bezerra A.L.F.M., Pinheiro E.B.F. (2021). Essential Oils as an Alternative for the Control of *Anopheles* Genus Larvae: A Review. Res. Soc. Dev..

[70] Gurunathan A., Senguttuvan J., Paulsamy S. (2016). Evaluation of mosquito repellent activity of isolated oleic acid, eicosyl ester from *Thalictrum javanicum*. Indian J. Pharm. Sci..

[71] Borges A.D.C., de Carvalho C.E.G., de Souza J.R.L., Morato E.F., Cadaxo-Sobrinho E.S., Marques D.D. (2021). Evaluation of the Chemical Composition and Larvicidal Activity of *Cymbopogon nardus* Essential Oil in the Control of *Aedes aegypti* in Southwestern Amazon. Holos.

[72] Benelli G., Rajeswary M., Vijayan P., Senthilmurugan S., Alharbi N.S., Kadaikunnan S., Govindarajan M. (2018). Essential Oil of *Boswellia ovalifoliolata* (Burseraceae) as an Ecological Larvicide? Toxicity Against Six Mosquito Vectors of Public Health Importance, Non-Target Mosquito Fishes, Back Swimmers, and Water Bugs. Environ. Sci. Pollut. Res..

[73] Ayinde A.A., Morakinyo O.M., Sridhar M.K.C. (2020). Repellency and larvicidal activities of Azadirachta indica seed oil on *Anopheles gambiae* in Nigeria. Heliyon.

[74] de Oliveira A.C., Simões R.C., Tavares C.P., Lima C.A., Sá I.S.C., da Silva F.M., Roque R.A. (2022). Toxicity of the essential oil from *Tetradenia riparia* (Hochstetter.) Codd (Lamiaceae) and its principal constituent against malaria and dengue vectors and non-target animals. Pestic. Biochem. Physiol..

[75] Simas N.K., Lima E.D.C., Conceição S.D.R., Kuster R.M., Oliveira Filho A.M.D., Lage C.L.S. (2004). Produtos naturais para o controle da transmissão da dengue: Atividade larvicida de *Myroxylon balsamum* (óleo vermelho) e de terpenóides e fenilpropanóides. Química Nova.

[76] Cruz I.L.S., Pimentel M.A.G., Nascimento T.A., Alves S.P., Maleck M., Queiroz M.M.C. (2024). Larvicidal activity and chemical composition of four essential oils against *Aedes aegypti* (Diptera: Culicidae). Braz. J. Biol..

[77] de Menezes Torres M.d.C., da Luz M.A., de Oliveira F.B., Barbosa A.J.C., de Araújo L.G. (2021). Chemical composition of essential oil from Croton *Heliotropiifolius kunth* (Euphorbiaceae) Leaves. Braz. J. Dev..

[78] Suppajariyawat P., Andrade A.F.B.D., Elie M., Baron M., Gonzalez-Rodriguez J. (2019). The use of chemical composition and additives to classify petrol and diesel using gas chromatography–mass spectrometry and chemometric analysis: A UK study. Open Chem..

[79] World Health Organization (WHO) (2005). Guidelines for laboratory and field testing of mosquito larvicides. World Health Organization Communicable Disease Control, Prevention and Eradication Who Pesticide Evaluation Scheme.

[80] Giatropoulos A., Kimbaris A., Michaelakis A., Papachristos D.P., Polissiou M.G., Emmanouel N. (2018). Chemical composition and assessment of larvicidal and repellent capacity of 14 Lamiaceae essential oils against *Aedes albopictus*. Parasitol. Res..

