# Supplementary material for: Larvicidal Potential of Trattinnickia Burserifolia Mart. Essential Oil in Controlling the Malaria Vector in the Amazon
Source: Pharmaceuticals (Basel). 2025 Apr 22;18(5):604. doi: 10.3390/ph18050604 (PMC12114282; doi:10.3390/ph18050604)
Supplement: Supplementary file 1 [file pharmaceuticals-18-00604-s001.zip › pharmaceuticals-3526966-supplementary.pdf]

The terpenes, which are the major constituents identified in *T. burserifolia* essential oil, have their spectrograms shown in Figures S1 to S5.

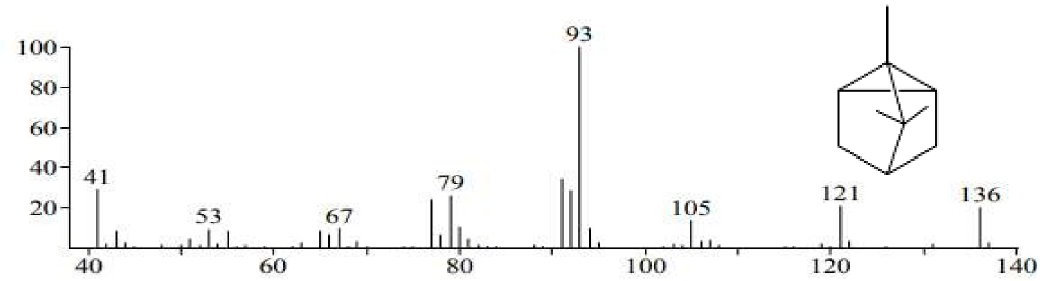

Figure S1. Tricyclene spectrum [46].

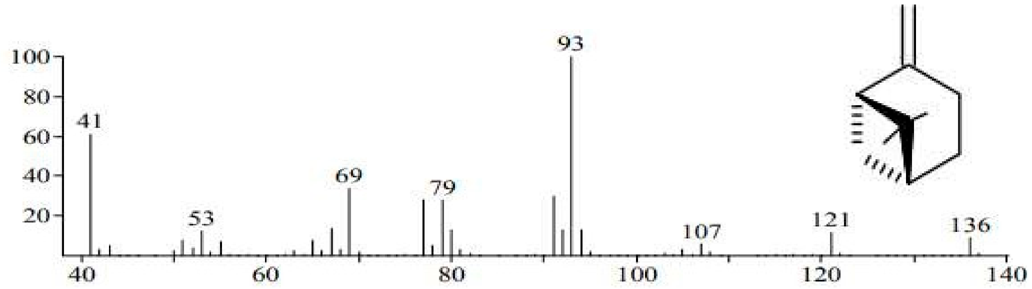

Figure S2. β-Pinene spectrum [46].

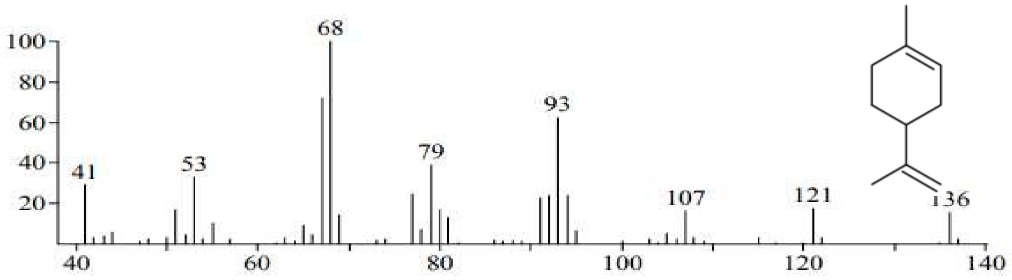

Figure S3. Limonene spectrum [46].

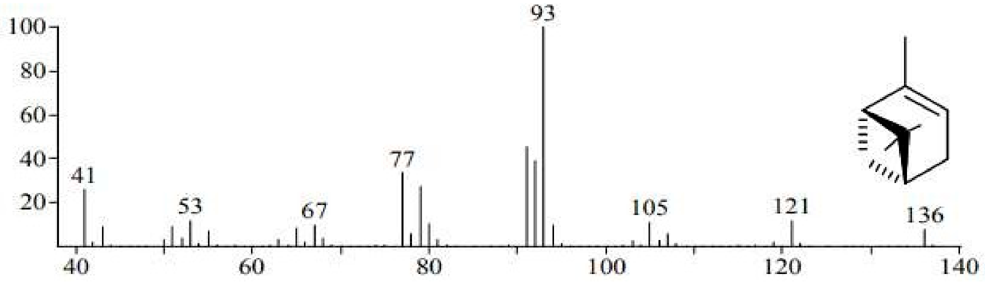

Figure S4. α-Pinene spectrum [46].

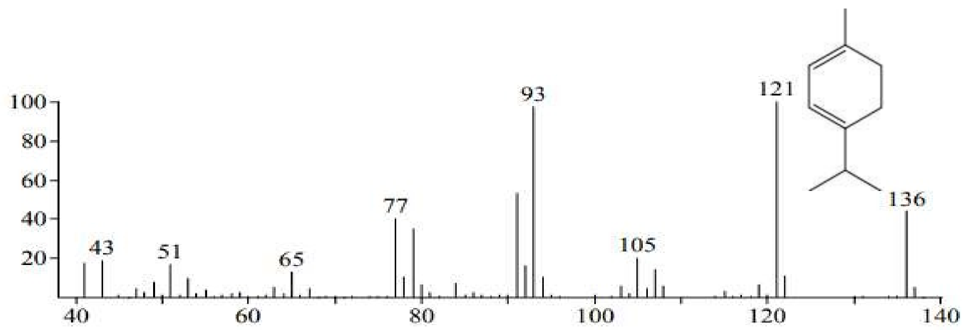

Figure S5. α-Terpinene spectrum [46].

Table S1. Publications found in scientific databases containing the terms 'Trattinnickia' and 'Malaria' between 2020 and 2024.

Research source	Article title	Reference
SciElo	Mechanical resistance and chemical composition of deteriorated Amazonian woods in field tests.	[26]
	Surface deterioration of five Amazonian woods exposed to natural weathering.	[27]
MedLine	Secondary Metabolites Found among the Species *Trattinnickia rhoifolia* Willd	[21]
	Acute Toxicity and Anti-Inflammatory Activity of *Trattinnickia rhoifolia* Willd (Sucuruba) Using the Zebrafish Model	[28]
Capes Periodical	Physicochemical Characterization and Thermal Behavior of Different Wood Species from the Amazon Biome	[29]
	Acute Toxicity and Anti-Inflammatory Activity of *Trattinnickia rhoifolia* Willd (Sucuruba) Using the Zebrafish Model	[28]
	Identification of flavonoids by LC-MS/MS in the leaf extract of *Trattinnickia rhoifolia* (Willd) and evaluation of antioxidant activity.	[30]
	A novel approach to maximize wood production in sustainable management of the Amazon rainforest	[31]

Google Scholar	Ethnobotanical treatment of tropical diseases, malaria and dengue, prescribed by Bioenergético practitioners and profile of the involved population in meridional amazon.	[23]
	General aspects of vegetation, flora, and useful plants from the Kanarakuni River valley, Upper Caura River, Bolívar State, Venezuela	[32]
	Amazonian Medicinal Smokes: Chemical Characterization of Burseraceae *Pitch oleoresin* (Breu) Combustion Products and Their Potential Therapeutic Application for Headache Relief	[22]
	Unveiling the leaf-dropping behavior behind bat folivory: Do bats employ biological control against roost parasites	[33]
	Phytochemistry and biological activities of amburana cearensis (alemão).	[34]
	Diversity of Plasmodium vectors and functional traits of trees in disturbed forests of Tingo María, 2022	[24]
